# miR-25-3p Modulates Tumor Aggressiveness and Ferroptosis Escape in T24 Bladder Cancer Cells In Vitro

**DOI:** 10.3390/ph18091382

**Published:** 2025-09-16

**Authors:** Andresa Hiromi Sakai, Érica Romão Pereira, Anna Gabriele Prado dos Santos, Débora Hipólito Quadreli, Luan Vitor Alves de Lima, Diego Luis Ribeiro, Samira Rahimirad, Carolina Mathias, Monyse de Nóbrega, Mário Sérgio Mantovani, Glaura Scantamburlo Alves Fernandes, Ilce Mara de Syllos Cólus, Juliana Mara Serpeloni

**Affiliations:** 1 Post-Graduation Program in Genetics and Molecular Biology, Department of General Biology, State University of Londrina, Londrina 86057-970, Brazil; andresa.hiromi@uel.br (A.H.S.); ericaa.romaopg@uel.br (É.R.P.); annagabriele.prado@uel.br (A.G.P.d.S.); luan.vitorlima@uel.br (L.V.A.d.L.); biomsm@uel.br (M.S.M.); glaura@uel.br (G.S.A.F.); colus@uel.br (I.M.d.S.C.); 2 Post-Graduation Program in Experimental Pathology, Department of Immunology, Parasitology and General Pathology, State University of Londrina, Londrina 86057-970, Brazil; debora.hipolito@uel.br; 3 Department of Microbiology, Institute of Biomedical Sciences, University of São Paulo, São Paulo 05508-900, Brazil; diegoluisribeiro@usp.br; 4 Urologic Oncology Research Group, Cancer Research Program, Research Institute of the McGill University Health Center (RI-MUHC), Montreal, QC H4A 3J1, Canada; samira.rahimirad@mail.mcgill.ca; 5 Post-Graduation Program in Genetics, Department of Genetics, Federal University of Parana, Curitiba 81530-980, Brazil; carolina.mathias@ufpr.br; 6 Cancer Research Program, Research Institute of the McGill University Health Centre, Montreal, QC H4A 3J1, Canada; monyse.denobrega@mail.mcgill.ca; 7 Department of Pathology, McGill University, Montreal, QC H4A 3J1, Canada

**Keywords:** ncRNAs, miR-106b/25, migration, oxidative stress, transfection, urothelial cancer

## Abstract

**Background/Objectives**: Urothelial bladder carcinoma (UBC) is one of the most prevalent malignancies worldwide, and efforts have intensified to identify molecular markers that improve the prognosis and reduce treatment costs. Among the regulators of tumor behavior, microRNAs (miRNAs) have emerged as promising biomarkers for cancer diagnoses and treatment. The modulation of miR-25-3p has been associated with pancreatic, colorectal, and lung cancers; its role in UBC remains poorly explored. In this study, we investigated the effects of miR-25-3p modulation in a high-grade and muscle-invasive bladder cancer (MIBC) cell line (T24), using in vitro functional assays and bioinformatics approaches. **Results:** Bioinformatics analyses using TCGA-BLCA datasets revealed that miR-25-3p is upregulated in tumor tissues compared to non-tumor tissues, prompting an investigation into its molecular targets and related pathways. The transfection of T24 cells with an miR-25-3p mimic and inhibitor led to respective overexpression (11.16-fold) and downregulation (-2.82-fold) compared to the negative control. Functionally, miR-25-3p overexpression increased cell proliferation, viability, and migration, while its inhibition decreased the cell migration capacity. A gene expression analysis revealed that miR-25-3p overexpression resulted in the downregulation of *TP53*, *AIFM1*, *NFE2L2*, *TFRC*, *ACSL4*, *SLC7A11*, and *SLC3A2*, whereas *MMP9*, *MMP11*, and *GPX4* were upregulated, suggesting a role in both migration and ferroptosis regulation. In the inhibitor group, increased *SLC3A2* and decreased *MMP11* expression further supported this connection. Our results using an in vitro model for MIBC with the transfection of T24 cells suggest that miR-25-3p influences key pathways involved in oxidative stress and cell death, promoting a more aggressive tumor phenotype. **Conclusions:** The modulation of miR-25-3p impacts the behavior of T24 bladder cancer cells and may indicate its role in disease progression. Our results underscore the potential of miR-25-3p as a prognostic biomarker and support further studies considering its therapeutic relevance in managing high-grade and muscle-invasive bladder cancer.

## 1. Introduction

Bladder cancer is one of the most commonly diagnosed cancers in the world and is ranked ninth for incidence rates in 2022, with approximately 614,000 new cases and 220,000 deaths reported. Men are affected more often than women. In men, this type of cancer ranks sixth for incidence (4.6%) and ninth for mortality (3.1%), with tobacco use being the primary risk factor [1]. Bladder cancer is characterized by a high recurrence rate, requiring patients to undergo long-term surveillance with invasive methods, especially those with aggressive neoplasms, which have a high mortality rate [2].

Urothelial bladder cancer (UBC) originates in the cells lining the urinary bladder tract, specifically the urothelial cells. Urothelial carcinomas include a group of neoplasms that affect the bladder, upper urinary tract (kidneys and ureters), and proximal urethra, with UBC accounting for 90–95% of cases [3]. UBC can be divided into (i) non-muscle-invasive (NMIBC), which is confined to the urothelium or lamina propria of the organ, and (ii) muscle-invasive (MIBC), when it invades the muscle layer. UBC tumors can also spread to other organs through metastases, especially in the lymph nodes, bones, lungs, liver, and peritoneum. Additionally, carcinoma in situ is a non-invasive, high-grade lesion with a high probability of recurrence and progression [4,5].

Therapeutic approaches differ based on the type of tumor that a patient has. For MIBC, the standard treatment is a radical cystectomy (RC) with the removal of the pelvic lymph nodes [6,7]. Although this approach is highly effective, the rates of recurrence, progression, and metastasis remain high, leading to a poor long-term prognosis for UBC patients. Therefore, discovering new biomarkers, targets, and therapeutic mechanisms for UBC treatment is essential for enhancing patient survival. Recently, microRNAs (miRNAs)—small non-coding RNAs (19–25 nucleotides) that regulate gene expression after transcription—have emerged as promising biomarkers. miRNAs function by binding to complementary sequences in the 3′ untranslated region (UTR) of target mRNAs, which results in translational repression or mRNA degradation [8]. Oncogenic miRNAs can suppress tumor suppressor genes or those involved in cell differentiation and apoptosis, thereby facilitating tumor progression. Conversely, tumor-suppressor miRNAs—often underexpressed in cancer—help inhibit tumor development [9,10].

The miR-106b/25 cluster, which includes miR-106b, miR-25, and miR-93, is highly conserved on chromosome 7q22, located in intron 13 of the *MCM7* gene (minichromosome maintenance complex 7). These miRNAs participate in various cellular processes, including proliferation, cell death (such as apoptosis and ferroptosis), differentiation, and metabolism, which are crucial for cellular development and function [11]. The deregulation of miR-25-3p has been associated with several diseases, including cancer, where it can act as an oncogene. Conversely, it also has the potential to serve as a tumor suppressor by targeting genes involved in tumor progression [12]. In UBC, previous studies have shown that miR-25 is involved in cell proliferation and tumor invasion by negatively regulating *PTEN*, suggesting that this miRNA may be a potential marker for UBC progression [13].

Understanding the regulatory mechanisms of miR-25-3p is essential for developing new targeted therapies and diagnostic methods, and for predicting prognoses. Therefore, this study investigated the biological role of miR-25-3p in the pathogenesis of UBC. We created a UBC in vitro model by transfecting an miR-25-3p mimic, its inhibitor, and a control miRNA into the invasive urothelial bladder carcinoma cell line T24. The transfection efficiency was analyzed along with the biological processes in the cancer cells, including the cell viability and death, proliferation, oxidative stress, migration, and changes in gene expression. Additionally, we used multiple bioinformatics tools to investigate the predicted targets of miR-25-3p, the biological pathways they regulated, and the gene expression patterns in TCGA samples with a low and high expression of this miRNA.

## 2. Results

### 2.1. In Silico Analysis

#### 2.1.1. Identification of miR-25-3p Target Genes

**Figure 1:**
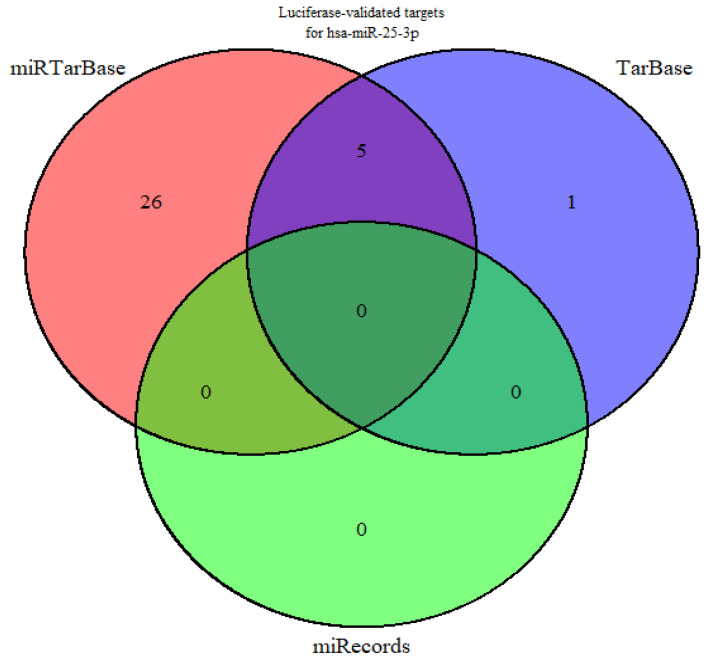
Venn diagram showing luciferase-validated target genes of hsa-miR-25-3p retrieved from miRTarBase, TarBase, and miRecords databases. No luciferase-validated targets were found in miRecords.

Using miRTarBase, miRecords, and TarBase, 32 genes were identified as validated targets of miR-25-3p through only luciferase assays (Supplementary Table S1). It was also observed that five validated target genes (*CDKN1C*, *BCL2L11*, *MDM2*, *FBXW7*, and *KAT2B*) overlapped across the databases, while 26 were found exclusively in miRTarBase and one in TarBase (Figure 1).

**Figure 2:**
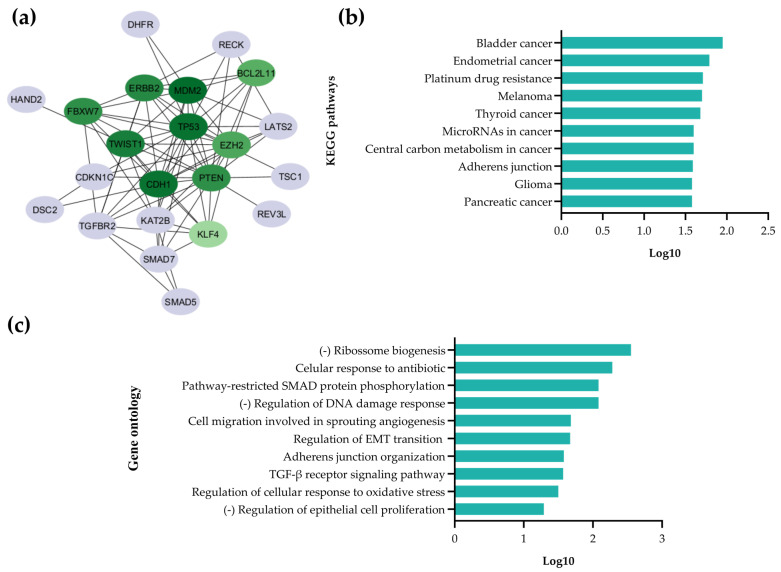
Proteins encoded by the validated target genes of miR-25-3p, the interaction network between them, and the cellular pathways modulated. (**a**) PPI network illustrating the interaction between miR-25-3p target proteins. Nodes represent individual proteins, and edges indicate protein–protein interactions. The MCC algorithm (cytoHubba) identified 10 hub proteins within the most densely connected regions. The scores are shown in a green scale, with darker colors indicating higher scores. (**b**) The Kyoto Encyclopedia of Genes and Genomes (KEGG) pathway enrichment analysis, highlighting key pathways associated with miR-25-3p target gene proteins. The x-axis displays the number of proteins annotated in each path (log10), while the y-axis lists the KEGG pathways. (**c**) The Gene Ontology (GO) enrichment analysis presents the top 10 enriched terms for each GO category: biological process (BP), cellular component (CC), and molecular function (MF). The x-axis indicates the number of proteins annotated in each term (log10) and the y-axis lists the GO terms.

To explore their biological roles, we conducted a protein–protein interaction (PPI) analysis using the STRING database, and nodes with isolated connections were excluded, resulting in a network of 22 nodes. The PPI network was visualized using Cytoscape (version 3.10.3) and the cytoHubba plugin, which identified the top 10 proteins using the MCC algorithm (Figure 2a). These proteins included p53 (tumor protein p53), MDM2 (MDM2 proto-oncogene), ERBB2 (Erb-b2 receptor tyrosine kinase 2), CDH1 (cadherin 1), TWIST1 (Twist family BHLH transcription factor 1), FBXW7 (F-box and WD repeat domain-containing 7), PTEN (phosphatase and tensin homolog), BCL2L11 (BCL2-like 11), EZH2 (enhancer of zeste 2 polycomb repressive complex 2 subunit), and KLF4 (Kruppel-like factor 4). TP53 (score = 0.999) and MDM2 (score = 0.999) emerged as the most significant hub proteins, highlighting their potential role in miR-25-3p-mediated regulation.

Kyoto Encyclopedia of Genes and Genomes (KEGG) and Gene Ontology (GO) analyses provided insight into the functional roles of the miR-25-3p target genes. The KEGG pathway analysis revealed that the validated targets of miR-25-3p are mainly enriched in bladder cancer, followed by endometrial cancer and platinum drug resistance (Figure 2b). Notably, the identified target genes correspond to key biological processes associated with UBC and cisplatin (cDDP) resistance, the primary chemotherapeutic agents used in UBC treatment, thereby reinforcing the relevance of miR-25-3p as a candidate for further investigation in the UBC context. GO enrichment highlighted biological processes such as ribosome biogenesis, the cellular response to antibiotics, and pathway-restricted SMAD protein phosphorylation (Figure 2c). Key pathways related to tumor progression, including epithelial cell migration, proliferation, oxidative stress, and cell–cell adhesion, were further explored in biological assays.

#### 2.1.2. Increased Expression of miR-25-3p in Bladder Cancer

**Figure 3:**
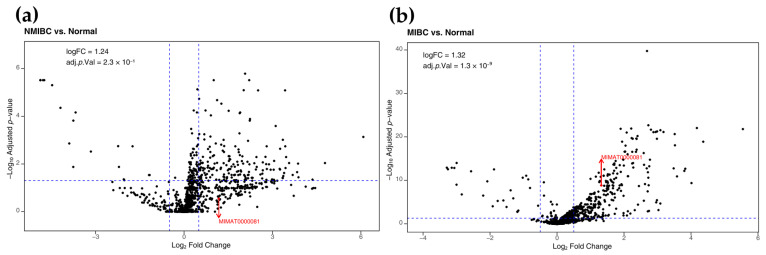
Volcano plot illustrating differentially expressed miRNAs in (**a**) non-muscle-invasive bladder cancer (NMIBC, N = 4) and (**b**) muscle-invasive bladder cancer (MIBC, N = 404) compared to normal tissues (N = 19), based on The Cancer Genome Atlas (TCGA) data. Each point represents an individual miRNA that is significantly upregulated (positive values) or downregulated (negative values). miR-25-3p, shown in red (indicated by the red arrow and the mature_mirna_acc code (MIMAT0000081), is overexpressed in MIBC tumors compared to normal tissues. The threshold lines (blue dashed lines) indicate the −0.5 <logFC> 0.5, and an adjusted *p*-value < 0.05.

To explore the miRNAs involved in bladder cancer progression, we performed differential expression analyses of miRNAs from “The Cancer Genome Atlas (TCGA)” database using bioinformatics tools. This analysis compared the miR25-3p expression in tumor tissues versus normal tissues in NMIBC (Figure 3a) and MIBC (Figure 3b). The results identified 538 and 424 miRNAs as differentially expressed in NMIBC and MIBC, respectively (*p* < 0.05). In MIBC, 278 miRNAs were upregulated and 25 were downregulated (*p* < 0.05). miR-25-3p was significantly overexpressed in MIBC (FC = 1.32, *p* = 1.3 × 10^−9^), while no significant differences were observed in the NMIBC group. Due to the small number of NMIBC cases in the TCGA dataset (N = 4), the statistical power for comparisons involving this subtype was considerably limited. Further studies with larger and more balanced cohorts are necessary to validate whether the expression of miR-25-3p differs between NMIBC and MIBC or is truly associated with invasive disease. However, despite its elevated expression, the miR-25-3p levels did not correlate with overall survival in patients with MIBC (Supplementary Figure S1).

### 2.2. Functional Assays in Invasive UBC T24 Cells

**Figure 4:**
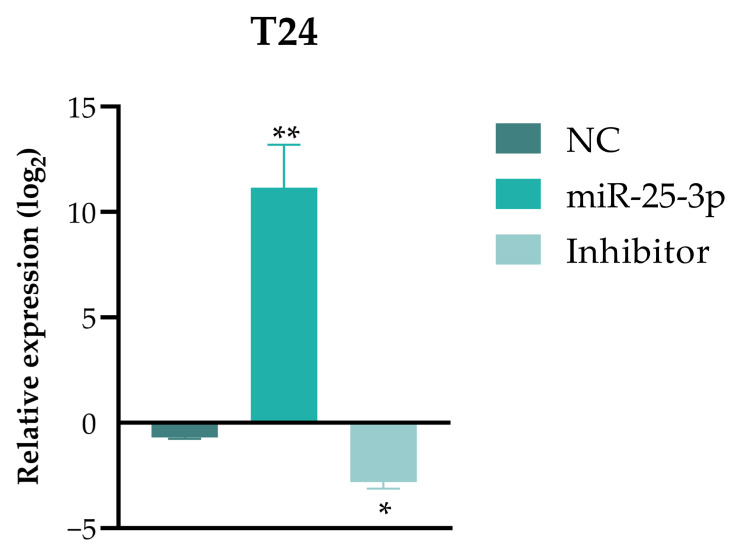
Real-time quantitative polymerase chain reaction analysis to assess miRNA mimic/inhibitor transfection efficiency in the T24 cell line after 48 h. The 2^−ΔΔCt^ method used the expression in the negative control group as a baseline reference. The miRNA RNU6 served as an endogenous control in each group (NC, miR-25-3p, and inhibitor). Each experiment was conducted in biological and technical triplicate. Significant differences from the control group (Student’s *t*-test): * *p* < 0.05, ** *p* < 0.01.

RT-qPCR confirmed the transfection efficiency. After 48 h, the miR-25-3p expression was significantly upregulated (11.16-fold) in the mimic (MI) group compared to the negative control (NC). In the inhibitor (IN) group, miR-25-3p was downregulated (−2.82-fold) (Figure 4). These results confirm the successful transfection of T24 cells, establishing 48 h as the optimal time point for subsequent in vitro functional assays.

### 2.3. Overexpression of miR-25-3p Increases Cell Proliferation and Plays a Role in Cell Viability

**Figure 5:**
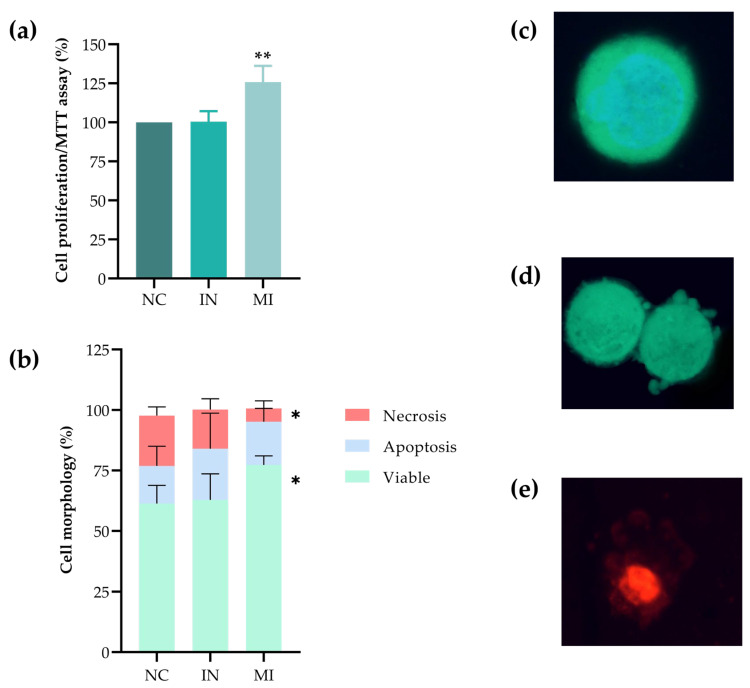
miR-25-3p regulates cellular proliferation and viability. (**a**) MTT assay measuring cell proliferation. (**b**) Quantification of viable, apoptotic, and necrotic cells based on morphological analysis. Cells were transfected for 48 h in both assays with miR-25-3p mimic (MI), inhibitor (IN), and a negative control (NC). (**c**–**e**) Representative images of viable (**c**), apoptotic (**d**), and necrotic (**e**) cell morphologies. The experiments were performed in three biological replicates (n = 3). The data are shown as the mean ± standard deviation. A one-way ANOVA followed by Dunnett’s post-test was used; * *p* < 0.05 and ** *p* < 0.01.

The MTT assay showed an increased cell proliferation in the MI group, suggesting that the overexpression of miR-25-3p promotes cell proliferation. In contrast, the IN group maintained proliferation levels similar to the NC (Figure 5a). In the cell death assay, the MI group exhibited a higher percentage of viable cells (FDA+ without apoptotic bodies) than the NC, along with a decrease in necrosis. The IN group exhibited a rate of viable cells comparable to that of the NC (Figure 5b), with a slight, but non-significant, increase in the percentage of apoptotic cells. The assay also differentiated between viable (Figure 5c), apoptotic (Figure 5d), and necrotic (Figure 5e) cells based on the presence of apoptotic bodies and propidium iodide (PI) staining in apoptotic and necrotic cells, respectively. Therefore, the overexpression of miR-25-3p would increase the proliferation and viability, while protecting T24 cells from death.

### 2.4. miR-25-3p Modulates the Redox Status

**Figure 6:**
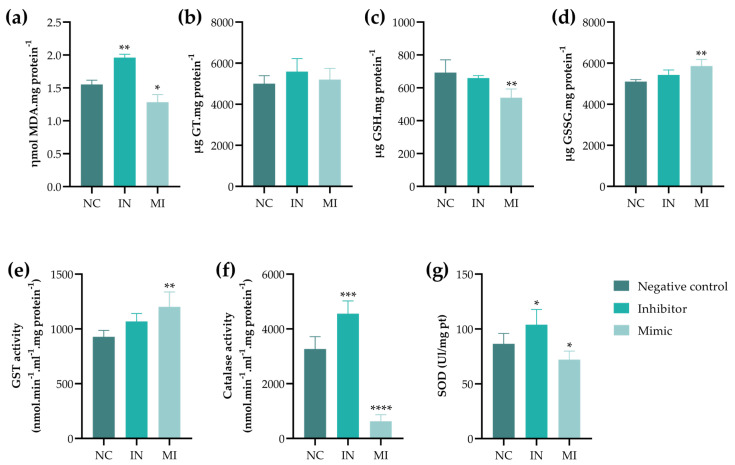
Regulation of cellular oxidative stress by miR-25-3p in T24 cells after 48 h of transfection with miR-25-3p mimic (MI), inhibitor (IN), and negative control (NC). (**a**) Measurement of lipid peroxidation by thiobarbituric acid-reactive substances (TBARSs). (**b**) Determination of total glutathione (GT) by Ellman’s reagent (DTNB) and nicotinamide-adenine dinucleotide phosphate (NADP). (**c**) Quantification of reduced glutathione (GSH) by DTNB. (**d**) Calculation of oxidized glutathione (GSSG) levels (GT-(GSH/2)). (**e**) Measurement of glutathione S-transferase (GST) activity by 1-chloro-2,4-dinitrobenzene (CDNB). (**f**) Measure of catalase activity by peroxide dismutation. (**g**) Measure of superoxide dismutase (SOD) activity. Values represent the mean ± standard deviation of three experiments performed in triplicate. A one-way ANOVA followed by Dunnett’s post-test was used; * *p* < 0.05, ** *p* < 0.01, *** *p* < 0.001, and **** *p* < 0.0001 compared to negative control (NC).

Oxidative stress assays were performed to investigate potential cell death pathways, including necroptosis and ferroptosis, given that the morphological analyses revealed features consistent with necrotic cell death (characterized by membrane rupture). The IN group exhibited increased lipid peroxidation, whereas the MI group showed a reduction in this marker (Figure 6a). Additionally, the MI group demonstrated a decrease in reduced glutathione (GSH) levels (Figure 6c), accompanied by an increase in oxidized glutathione (GSSG) levels (Figure 6d) and enhanced glutathione S-transferase (GST) activity (Figure 6e). Antioxidant enzyme activities—specifically catalase (CAT) and superoxide dismutase (SOD)—were elevated in the absence of miR-25-3p (IN group), while their activities were reduced in the presence of this miRNA (MI group) (Figure 6f–g).

### 2.5. miR-25-3p Modulates T24 Cell Migration

**Figure 7:**
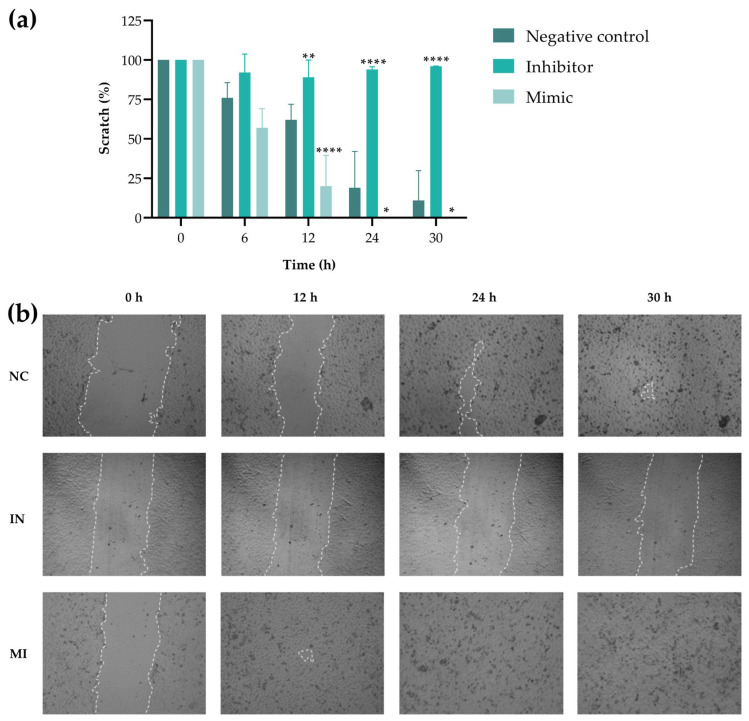
miR-25-3p (mimic/inhibitor) modulates T24 horizontal cell migration. (**a**) T24 cells transfected with the miR-25-3p mimic (MI), inhibitor (IN), or negative control (NC) were subjected to a wound healing assay, with images captured at 0, 6, 12, 24, and 30 h. (**b**) Representative images of the wound healing assay at 0, 12, 24, and 30 h for the negative control (NC), inhibitor (IN), and mimic (MI) groups. The wound size was measured using the ImageJ/Fiji^®^ software 9 (version 1.54). All the experiments were performed in biological and technical triplicate (n = 3). Values represent the mean ± standard deviation. A one-way ANOVA followed by Dunnett’s post-test was used; * *p* < 0.05, ** *p* < 0.01, and **** *p* < 0.0001.

The wound healing assay verified the effect of miR-25-3p on T24 cell migration. The results showed that the MI group migrated significantly faster than the NC group, beginning at 12 h and completing wound closure within 24 h (Figure 7a). Furthermore, in the IN group, the horizontal migration of T24 cells was much slower, and the wound remained open after 30 h. Figure 7b shows representative images of the test.

### 2.6. Gene Expression Showed That miR-25-3p Modulates Ferroptosis and MMPs Pathways

**Figure 8:**
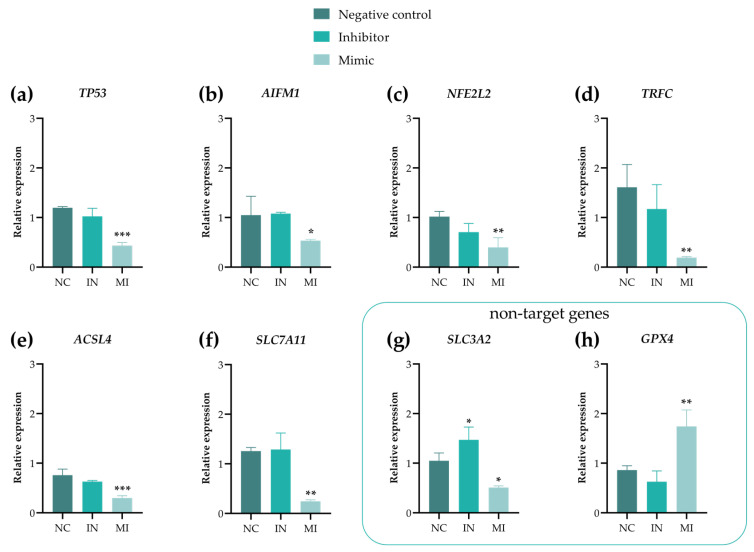
Real-time quantitative polymerase chain reaction analysis of genes associated with oxidative stress and cell death (apoptosis and ferroptosis). (**a**) *TP53*, (**b**) *AIFM1*, (**c**) *NFE2L2*, (**d**) *TRFC*, (**e**) *ACSL4*, (**f**) *SLC7A11*, (**g**) *SLC3A2*, and (**h**) *GPX4*. Gene expression was observed in the NC (negative control), IN (inhibitor) group, and MI (mimic) group in T24 cells after 48 h of transfection. The mRNA levels of the genes were normalized to *β-actin*. Student’s t-test was conducted to perform a statistical analysis, with the data presented as the mean ± standard deviation of three biological replicates (N = 3). * *p* < 0.05, ** *p* < 0.01, and *** *p* <0.001. *TP53*: tumor protein p53; *AIFM1*: apoptosis-inducing factor mitochondria-associated 1; *NFE2L2:* nuclear factor erythroid 2-related factor 2; *TRCF*: transferrin receptor; *ACSL4*: acyl-CoA synthetase long-chain family member 4; *SLC7A11*: solute carrier family 7 member 11. *SLC3A2*: solute carrier family 3 member 2. *GPX4:* glutathione peroxidase 4.

We validated the expression of six predicted miR-25-3p target genes—*TP53*, *AIFM1*, *NFE2L2*, *TRFC*, *ACSL4*, and *SLC7A11*—using RT-qPCR. Additionally, other genes were chosen to explore the viability, oxidative stress, and antimigratory effects observed in functional assays. The overexpression of miR-25-3p (MI) downregulated all the target genes, whereas the IN group maintained expression levels similar to those of the NC (Figure 8a–f). The non-target genes (*GPX4*, *MMP9*, and *MMP11*) were overexpressed in the MI group, except for *SLC3A2*.

Given the increased proliferation and viability observed in the functional assays, we examined the role of miR-25-3p in ferroptosis-related pathways. Ferroptotic cell death aligns with membrane disruption (linked to the necrotic shape seen in the cell death test) and a redox imbalance (shown by oxidative stress markers and *NEF2L2* downregulation). Using the databases miRTarBase, miRecords, and TarBase to identify the potential target genes of miR-25-3p, we found several genes associated with ferroptosis, including *TFRC*, *ACSL4*, and *SLC7A11*. A gene expression analysis revealed that all these targets were downregulated upon the overexpression of miR-25-3p (Figure 8d–f). Furthermore, a bioinformatics analysis of TCGA patient samples showed a negative correlation between *SLC3A2* and miR-25 expression (see Table S2). Although *SLC3A2* has not been identified as a direct target of miR-25-3p in databases, our data show that it was downregulated in the presence of miR-25-3p (MI group) and upregulated in its absence (IN group) (Figure 8g). Additionally, the MI group showed increased *GPX4* expression (Figure 8h).

**Figure 9:**
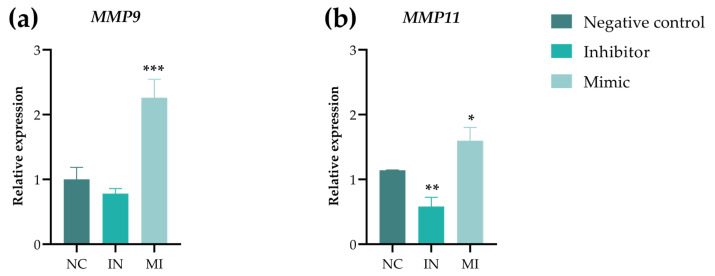
Real-time quantitative polymerase chain reaction analysis of genes related to cell migration: (**a**) *MMP9* and (**b**) *MMP11*. All genes were examined in the NC (negative control), IN (inhibitor) group, and MI (mimic) group in T24 cells after 48 h of transfection. The mRNA expression levels were normalized to the *β-actin* levels. Student’s t-test was used for the statistical analysis, with the data shown as the mean ± standard deviation of three biological replicates (n = 3). * *p* < 0.05, ** *p* < 0.01, and *** *p* < 0.001. *MMP9*: matrix metallopeptidase 9; *MMP11:* matrix metallopeptidase 11.

In addition, we examined the antimigratory effects by analyzing the modulation of metalloproteinase (MMP) gene expression, specifically *MMP9* (gelatinase) and *MMP11* (stromelysin) (Figure 9a,b). The MI group showed an increased expression of both MMPs, whereas the IN group showed a reduction only in *MMP11*. These results indicate that miR-25-3p influences MMP expression during T24 cell migration.

## 3. Discussion

Recently, several studies have identified the important role of miR-25-3p in various cancers; however, the specific role of this miRNA in UBC and its associated mechanisms remain unclear. A KEGG pathway enrichment analysis of the predicted target genes for miR-25-3p highlighted key pathways related to bladder cancer and platinum resistance, emphasizing the importance of this miRNA in the context of UBC. GO demonstrated its essential role in cancer-related cellular pathways, including cell migration, proliferation, adhesion, and oxidative stress. After transfecting UBC T24 cells, the overexpression of miR-25-3p increased cell proliferation, viability, and migration, and it regulated ferroptosis by modulating the redox status and causing oxidative stress. In contrast, downregulating miR-25-3p suppressed cell migration and elevated the antioxidant enzymatic levels.

Differential miRNA expression can be considered a hallmark of tumorigenesis and has attracted extensive research interest due to its potential as a cancer biomarker and therapeutic target [14]. The relevance of our results in the context of UBC is that the analysis of TCGA samples for miRNA expression revealed several differentially expressed miRNAs in bladder cancer, including miR-25-3p. Additionally, the KEGG pathway analysis emphasized the importance of miR-25-3p target genes in bladder cancer.

The increase in cell proliferation observed in the MTT assay in the MI group is similar to the results seen in different cancers with the overexpression of miR-25-3p, such as cholangiocarcinoma [15], pancreatic cancer [16], and gastric cancer [17]. Li et al. [18] demonstrated that miR-25-3p overexpression significantly promoted liver cell proliferation in vitro, as measured by the CCK-8 assay. Additionally, the authors showed that miR-25-3p overexpression resulted in the downregulation of apoptosis-associated proteins, thereby promoting enhanced cell growth and reducing apoptotic activity. Our findings also support previous research, which observed an increase in viable cells (FDA+ without apoptotic bodies) in the MI group using the morphologic assay for cell death.

Although studies on miR-25-3p in the context of oxidative stress and tumor progression are limited, this study provides valuable insights into its regulatory role in antioxidant pathways during tumor development. The results indicate that miR-25-3p overexpression may suppress CAT and SOD activity. Additionally, a decrease in TBARS levels, along with the reduction in reduced glutathione (GSH) and the increase in oxidized glutathione (GSSG), suggests that the consumption of GSH and its conversion to GSSG reflect an antioxidant response to neutralize reactive oxygen species. Furthermore, the decrease in GSH levels combined with increased GST activity suggests a possible chemoresistance mechanism via enhanced drug efflux [19]. This may indicate the enhanced conjugation of GSH to reactive lipid intermediates, contributing to the reduction in lipid peroxidation [20,21]. Meanwhile, the IN group exhibited increased lipid peroxidation, which may have contributed to the heightened activity of antioxidant enzymes.

The modulation of miR-25-3p expression also affected the cellular migratory capacity. The migration and invasion of tumor cells through the extracellular matrix are related to multiple biochemical and morphological changes during the epithelial–mesenchymal transition (EMT), which promotes cancer progression and metastasis [22]. Here, we evaluated T24 cell migration using a wound healing assay, and the control group closed the wound within 24 h. This high migratory activity of T24 cells has been previously demonstrated [23,24,25] and reflects their invasive characteristic. Our results showed an increase in migration in the MI group and a decrease in the IN group. This modulation was previously observed in T24 (IN group) and 5637 (MI group) cells transfected with miR-25 [13]. Additionally, the effect of modulating the miR-25-3p expression on cell migration has been demonstrated in vitro for other cancer models, including models of gastrointestinal cancer [26], lung cancer [27], hepatocellular carcinoma [28,29], and colorectal cancer [30]. Our findings indicate that this miRNA may also play a significant role in bladder cancer progression.

mir-25-3p has several target genes related to the regulation of cell proliferation, death, and migration. *TP53*, a significant direct target of miR-25-3p, was downregulated in the MI group, likely contributing to the increased proliferation and survival of UBC T24 cells. This downregulation may promote cell cycle progression by preventing arrest, inhibiting apoptosis, and potentially suppressing ferroptosis, collectively encouraging tumor cell survival [31,32]. Furthermore, the increased cell viability in the MI group may also be associated with the absence of apoptotic cell death due to the downregulation of *AIFM1*, a key protein involved in caspase-independent cell death [33]. A decrease in necrotic cell death was also observed in the MI group. Alongside the oxidative stress results, this indicates a potential role of miR-25-3p in regulating cell death via ferroptosis, which occurs in conjunction with oxidative stress and membrane rupture. Ferroptosis plays a pivotal role in bladder cancer metastasis, the treatment response, and the prognosis. Identifying ferroptosis-related molecular biomarkers enables more accurate prognostic stratification and the development of targeted therapies [34,35].

We identified several genes as predicted targets of miR-25-3p through an in silico analysis using the miRTarBase, miRecords, and TarBase databases. Although the downregulation of *NFE2L2* observed in the MI group could potentially induce ferroptosis [36], the expression profile of other genes related to this cell death pathway suggests that ferroptosis might be inhibited. The downregulation of *TFRC* leads to reduced iron transport [37], and the low expression of *ACSL4* impairs the catalysis of the substrates necessary for the formation of lipid hydroperoxides [38], thereby reducing ferroptosis-mediated cell death in tumor cells. Additionally, we assessed the expression of genes involved in the GPX4 signaling pathway. While *SLC7A11* and *SLC3A2*—responsible for cystine transport and subsequent GPX4 activation [39]—were downregulated by miR-25-3p, *GPX4* expression itself was increased, suggesting an alternative activation route for this gene and, consequently, the potential activation of the GPX4 pathway, ultimately inhibiting ferroptosis.

To better understand ferroptosis, a bioinformatics analysis was performed using TCGA-BLCA samples to identify potential mRNAs linked to ferroptosis and increased miR-25-3p expression (see Table S2). This analysis showed the upregulation of *SLC7A10*, which directly facilitates cystine uptake, leading to GPX4 activation and a subsequent decrease in ferroptosis [40]. However, a limitation of our study was that it did not include specific ferroptosis modulators, such as erastin or RSL3. Further studies are warranted to validate the involvement of ferroptosis in this context functionally.

We evaluated the increase in MMP expression, previously reported in the literature, as a possible pathway modulated by miR-25-3p [41]. In UBC T24 cells, the downregulation of *MMP9* and *MMP11* was clearly correlated with the results from the migration assay. MMPs are divided into five families, one of which is the gelatinases, to which *MMP9* belongs. This enzyme degrades various components of the extracellular matrix (ECM), including elastin and collagen, thereby promoting processes such as cell proliferation, migration, and angiogenesis [42]. *MMP11* (stromelysin-3) belongs to the stromelysin subfamily and is primarily involved in cleaving regulatory proteins, such as the alpha-1 protease inhibitor, and in tissue remodeling [43]. This metalloproteinase is activated inside cells and secreted in its active form. It can be highly expressed by both stromal cells—especially tumor-associated fibroblasts—and tumor cells themselves, aiding in tumor invasion, cell survival, and immune evasion [44].

Our results, obtained by downregulating the *TP53* gene, align with those of metalloproteinases. Although p53 primarily regulates the cell cycle, repairs DNA damage, and triggers apoptosis, its reduced expression is also associated with increased migratory and invasive abilities of tumor cells [45]. In this context, p53 functions as a metastasis suppressor by decreasing the expression of matrix metalloproteinases [46].

Furthermore, the expression of *MMP9* and *MMP11* has been linked to the TGF-β1-signaling pathway, where the phosphorylation of SMAD2/3 promotes the transcription of these two metalloproteinases [47,48]. This pathway is particularly relevant in the context of miR-25-3p because SMAD2/3 activation requires phosphorylation, which can be inhibited by *SMAD7*, thus preventing MMP expression [49]. Among the validated targets of miR-25-3p, *SMAD7* is notable; therefore, the overexpression of this miRNA may inhibit *SMAD7*, leading to the activation of the SMAD2/3 pathway, the increased transcription of *MMP9* and *MMP11,* and enhanced cell migration, as seen in the MI group. Conversely, in the IN group, cell migration was inhibited, along with reduced *MMP11* expression, which may be attributed to the presence of *SMAD7* resulting from the absence of miR-25-3p.

**Figure 10:**
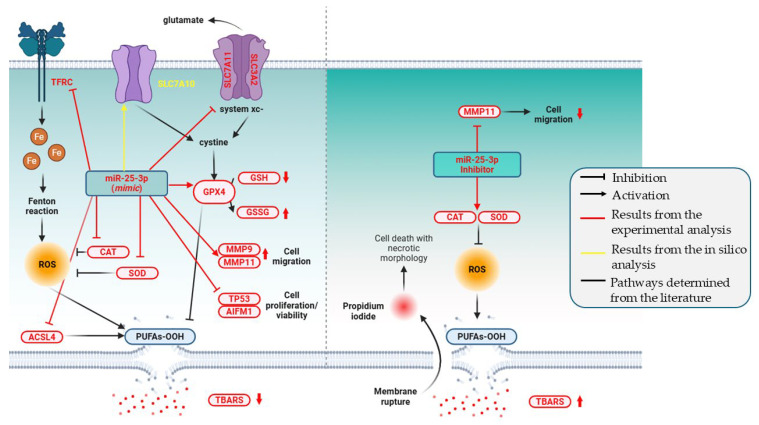
Schematic representation of the proposed mechanism of miR-25-3p in T24 bladder cancer cells. The overexpression of miR-25-3p (mimic) led to the inhibition of CAT and SOD activities, resulting in an initial accumulation of ROS. However, the decrease in GSH and increase in GSSG indicate a compensatory mechanism via the activation of GPX4, which contributes to the reduction in TBARS levels. In addition, the downregulation of TFRC and ACSL4 may further decrease lipid peroxidation. The downregulation of SLC7A11 and SLC3A2 suggests an alternative compensatory pathway for GPX4 activation, through direct cysteine import mediated by SLC7A10. Moreover, miR-25-3p enhanced the cell migratory capacity through the upregulation of MMP9 and MMP11, and promoted proliferation and viability via the downregulation of TP53 and AIFM1. Conversely, the inhibition of miR-25-3p (inhibitor) increased the TBARS concentrations, despite higher levels of CAT and SOD activities, leading to cell death with necrotic morphology (ferroptosis), as evidenced by the propidium iodide uptake. The inhibitor treatment also decreased the migratory capacity of T24 cells through the downregulation of MMP11.

To integrate our findings, we present a concluding schematic (Figure 10) summarizing the effects of miR-25-3p in T24 bladder cancer cells. This figure depicts its role in modulating oxidative stress and ferroptosis-related pathways, while also impacting tumor-associated processes such as proliferation and migration. It consolidates biochemical alterations (CAT, SOD, GSH/GSSG, TBARS), transcriptional changes in oxidative stress and ferroptosis-related genes (*GPX4*, *TFRC*, *ACSL4*, *SLC7A11/SLC3A2*, *SLC7A10*), and functional outcomes (cell viability, necrotic morphology, MMP-driven migration). Collectively, the schematic underscores miR-25-3p as a central regulator of ferroptosis evasion and tumor progression in bladder cancer, reinforcing the key conclusions of this study.

Our results are pioneering in suggesting the role of mR-25-3p in the modulation of ferroptosis in T24 cells cultured in vitro. However, the present study has some limitations, which were not the objective of this manuscript, but that may guide future research on miR-25-3p in the context of UBC, including the following: (i) the use of a single bladder cancer cell line, which limits the generalizability of the findings; (ii) the need for more comprehensive analyses employing high-throughput approaches; (iii) validation through in vivo assays using animal models, such as xenograft tumors; and (iv) an assessment of the miR-25-3p levels in liquid biopsies from UBC patients.

## 4. Materials and Methods

### 4.1. Bioinformatic Analysis

The STRING online database generated protein–protein interaction (PPI) networks [50]. The interaction scores between protein pairs were exported from STRING in .tsv format and utilized to create correlation matrices with the “ggplot2” package in R. Hub proteins, representing the top 10 proteins with the highest degree of connectivity within the PPI network, were identified using the CytoHubba plugin in Cytoscape [51]. To further explore miR-25-3p-related signaling pathways, enrichment analyses were conducted using the KEGG and GO databases. We obtained miRNA expression and clinical outcome data for 404 bladder cancer tumors and 19 normal tissues from The Cancer Genome Atlas (TCGA) via the GDC Data Portal (https://portal.gdc.cancer.gov/, accessed 4 February 2025). Differential expression analyses and visualizations, including volcano plots, were conducted using R (version 4.3.2), a programming language and software environment for statistical computing and data visualization.

#### 4.1.1. Selection of Putative Targets Regulated by miR-25-3p

Computational predictions of the miR-25-3p target genes were evaluated using miRTarBase, miRecords, and TarBase. These three databases collectively identified 1524 genes with binding sequences for the miR-25-3p seed region. Of these, 32 targets have been experimentally validated through luciferase reporter assays (Supplementary Table S1). The proteins codified by these 32 genes were used to generate protein–protein interaction (PPI) networks using STRING.

#### 4.1.2. Survival Analysis

To evaluate the impact of the differential expression of miR-25-3p target genes on patient survival, data for the miR-25-3p expression were retrieved from TCGA using the Bioconductor package. Kaplan–Meier (KM) survival curves were generated in R to illustrate the relationship between the gene expression and patient outcomes. Bladder cancer patients were stratified into high- and low-expression groups based on their median miRNA levels, and statistical significance was determined using the log-rank test. We collected the data on 4 February 2025, and archived all the analysis outputs and R scripts for future studies.

#### 4.1.3. Analysis of miRNA–mRNA Association of Ferroptosis-Related Genes

The association between miR-25-3p and the expression of ferroptosis-related genes was investigated using the approach described by Jacobsen et al. [52], based on the TCGA-BLCA. Only miRNA–mRNA pairs located on different chromosomes or more than 10 Mb apart on the same chromosome were considered to avoid potential *cis*-regulatory bias. Associations were modeled using multivariate linear regression according to the following formula: mRNA expression (log_2_) ~ miRNA expression (log_2_) + DNA methylation (β) + DNA copy number (log_2_). The data were preprocessed as follows: the mRNA expression values were quantified in TPM and log_2_-transformed after adding a pseudocount; the miRNA expression was also log_2_-transformed with a pseudocount; and missing methylation values were imputed using the probe-wise median. The *p*-values were adjusted for multiple comparisons using the false discovery rate (FDR) method.

### 4.2. Cell Line and Cultivation Conditions

The T24 cell line (ATCC^®^ HTB-4), derived from grade III transitional cell urothelial carcinoma of the bladder, was generously provided by Dr. Priscyla Daniely Marcato Gaspari (Faculty of Pharmaceutical Sciences of Ribeirao Preto, University of São Paulo). In culture, T24 cells were cultured in Roswell Park Memorial Institute 1640 (RPMI 1640) medium (11875093, Gibco, Grand Island, NE, USA) supplemented with 10% sterile triple-filtered fetal bovine serum (FBS) (10-bio500, Nova Biotecnologia, São Paulo, Brazil) and 1% antibiotic–antimycotic solution (100X; 15240062, Gibco, USA). The cells were incubated in a water-jacketed incubator (3110 Series II; Thermo Fisher Scientific, Grand Island, NE, USA) at 37 °C with 5% CO_2_ and 96% relative humidity. The Paternity Investigation Program confirmed the authenticity of the cell line through a DNA analysis (PIPAD), coordinated by Dr. Karen Brajão de Oliveira from the State University of Londrina, using short tandem repeat (STR) genotyping (Figure S1) with the PowerPlex^®^ Fusion 6C System (Promega São Paulo, Brazil) and the ABI 3500—Applied Biosystems. It used a 26 STR loci panel with (D21S11, D3S1358, D7S820, D1S1656, D5S818, D2S441, TPOX, D10S1248, D8S1179, D13S317, D12S391, Penta E, D19S433, D16S539, SE33, D18S51, D22S1045, D2S1338, DYS391, CSF1PO, FGA, Penta D, DYS576, TH01, DYS570, and WA) plus gender determination (AMEL) (Supplementary Figure S2).

### 4.3. Transfection of T24 Cells with miR-25-3p, miRNA Extraction, and RT-qPCR

T24 cells were transfected with the following miR-25-3p oligonucleotides: mirVana^®^ miRNA mimic (4464066, #MC10584, Ambion, Austin, TX, USA), mirVana^®^ miRNA inhibitor (4464084, #MH10584, Ambion, Austin, TX, USA), and negative control #1 (4464058, Ambion, Austin, TX, USA), a random-sequence miRNA mimic validated in human cells and tissues, with no detectable impact on endogenous miRNA function. The final concentrations were 10 μM for the mimic and negative control and 50 μM for the inhibitor, using Lipofectamine 2000 (11668-027, Invitrogen, Carlsbad, NM, USA). Opti-MEM Reduced Serum Media (31985070, Thermo Fisher Scientific, Grand Island, NE, USA) was used for a better transfection efficiency. The T24 transfected cells were incubated in the antibiotic-free medium at 37 °C and 5% CO_2_ for 48 h, as previously standardized [53], to assess the gene expression and perform functional assays.

Small-RNA extraction was performed using the miRNeasy Mini Kit (217004, Qiagen, Hilden, Germany) according to the manufacturer’s instructions. The RNA concentration and purity were measured with a NanoDrop 2000 spectrophotometer (Thermo Fisher Scientific, USA). For expression profiling, 1.4 ng of miRNA was used for reverse transcription. For cDNA synthesis, the miRNA was reverse-transcribed, and the resulting cDNA was diluted by 1:4 (*v*/*v*; μL) before use in the qPCR reaction. RT-qPCR was performed on the Applied Biosystems™ StepOnePlus™ Real-Time PCR System (Thermo Fisher Scientific, Singapore) (with the following components and cycling conditions: 5.5 μL of 2X TaqMan Universal PCR Master Mix (4440038, Applied Biosystems, Foster City, CA, USA), 0.45 μL of miRNA-specific TaqMan probe (4427975, Applied Biosystems, USA), and 7 μL of cDNA (extracted from the miRNA), diluted. The cycling conditions were as follows: 50 °C for 2 min, 95 °C for 10 min, and 50 cycles at 95 °C for 15 s and 60 °C for 1 min. The constitutive miRNA RNU6 was used as an endogenous control for normalization. Based on an miRNA expression analysis, the optimal time for transfection and subsequent functional assays was 48 h post-transfection.

### 4.4. Preparation of Monolayer Cell Culture (2D)

All the assays, except for cell migration, were performed in 96-well plates with 1 × 10^4^ cells per well. The cells were then stabilized for 24 h in RPMI 1640 culture medium supplemented with 10% FBS. After stabilization, the culture medium was removed, and the cells were washed with phosphate-buffered saline (PBS). As previously established, the transfection solution containing miR-25-3p was added to fresh medium with FBS without antibiotics. UBC T24 cells were transfected for 48 h before all of the functional assays, except for wound healing, which was performed during transfection.

### 4.5. MTT Assay

Cell proliferation was measured using FBS in the MTT assay (3-(4,5-dimethylthiazol-2yl)-2,5-diphenyltetrazoline bromide) (cat. No M6494; Thermo Fisher Scientific, Eugene, OR, USA), and following the method described by Mosman et al. [54]. After transfection, the MTT solution (0.50 mg/mL final concentration) was added to the wells and incubated for 4 h. The formazan crystals were solubilized in dimethyl sulfoxide (DMSO), and the absorbance was measured at ʎ = 540 nm using a microplate spectrophotometer (Biotek Eon, Winooski, VT, USA). The absorbance values were normalized by setting the negative control, considered as 100% cell viability, and the results were expressed as a percentage (%) of viable cells.

### 4.6. Morphological Assay (Triple Staining) for Cell Death Assessment

A cell death analysis was performed using the triple staining method [55]. After 48 h of transfection, the culture medium was removed, and the cells were washed with 1X PBS. Then, they were trypsinized for 3–5 min using TrypLE Express (cat. No. 12605028; Thermo Fisher Scientific, Grand Island, NE, USA). The cell suspension, previously removed medium, and PBS wash were centrifuged at 2500 rpm for 5 min, and the supernatant was discarded. A triple-stained fluorochrome mixture was prepared using PI, Hoescht 33342 (HO), and fluorescein diacetate (FDA) dyes. For the analysis, 30 μL of the homogenized suspension was mixed with 6 μL of the fluorochrome mixture (final concentrations: 4.0 μg/mL HO; 7.5 μg/mL FDA; 1.0 μg/mL PI). Approximately 200 cells per slide were analyzed using an Olympus BX43 fluorescence microscope (Olympus Microscopy, Europe) with a 40× objective. The following cell types were identified based on their fluorescence: (i) viable cells (FDA+, without apoptotic bodies), (ii) necrotic cells (FDA-; PI+), (iii) early apoptotic cells (FDA+; PI- and presence of apoptotic bodies) and (iv) late apoptotic cells (FDA+; PI+ and apoptotic bodies).

### 4.7. Wound Healing (Scratch Assay)

The wound healing assay was performed in 24-well plates with 1.1 × 10^5^ cells per well, incubated for 24 h to form a confluent monolayer. A “scratch” was created using a 200 μL pipette tip, and the plate was carefully washed with PBS to remove detached cells. The miR-25-3p transfection solutions were added, and the wells were filled with antibiotic-free culture medium. Images were taken at 0, 6, 12, 24, and 30 h after transfection and analyzed with the ImageJ/Fiji^®^ software 9 (version 1.54) using the *Wound_healing_size_tool* plugin [56]. Wound closure was expressed as a percentage, with the 0 h time point set as 100% of the wound size.

### 4.8. Oxidative Stress

Protein quantification was conducted using the Bradford method [57], a step in normalizing data for oxidative stress studies. A standard curve was created with serial dilutions of bovine serum albumin (BSA) ranging from 0.06 to 1.0 mg/mL. The samples and standards were pipetted in triplicate into a 96-well microplate, followed by the addition of 250 µL of Bradford reagent. After 5 min of incubation protected from light, the absorbance was measured at 595 nm using a microplate spectrophotometer. The protein concentrations were determined from the standard curve, and all the samples were then normalized to a final concentration of 1.0 mg/mL before use in downstream-specific assays. Before all the tests, the cells were transfected for 48h with the MI, IN, or NC.

#### 4.8.1. Quantification of TBARS

The thiobarbituric acid reactive substance (TBARS) method was employed to investigate whether miR-25-3p contributes to lipid peroxidation. For this purpose, 50 μL of each sample from the NC, IN, and MI groups were pipetted into a 96-well plate, followed by the addition of 5 μL of FeCl3, 5 μL of ascorbic acid, 50 μL of trichloroacetic acid (TCA), and 50 μL of thiobarbituric acid (TBA), and left in a water bath at 90 °C for 15 min. After this time, the reaction was stopped by placing the plate on an ice surface. The measurement was performed at *λ*= 535 and *λ* = 572 nm [58].

#### 4.8.2. Assessment of GT, GSH, and GSSG Concentrations

The quantification of the total glutathione (GT) and reduced glutathione (GSH) was performed according to Rahman et al. [59] with modifications. The GT was determined using 5,5’-dithiobis-20-nitrobenzoic acid (DTNB), nicotinamide adenine dinucleotide phosphate (NADPH), and glutathione reductase (GR) on each sample. The GT concentrations were measured at *λ* = 412 nm after 15 min of incubation of the samples with the reaction medium. To determine GSH, DTNB was used in the homogenate, as evidenced by the formation of a yellow color. The GSH content was measured at **λ** = 412 nm. The quantification of oxidized glutathione (GSSG) was calculated according to the proposal of Carrara et al. [60], which takes into account the stoichiometry of the reaction determined by the following equation: GSSG (µM) = GT − (GSH/2).

#### 4.8.3. GST Activity

The activity of glutathione S-transferase (GST) was measured with the addition of 1-chloro-2,4-dinitrobenzene (CDNB) and a potassium phosphate buffer. The absorbance of the samples was measured at *λ* = 340 nm for 160 s, with 5 measurements taken at 40 s intervals [61].

#### 4.8.4. CAT Activity

The enzymatic activity of catalase (CAT) was determined by measuring the degradation of hydrogen peroxide in the presence of oxygen and water. After selecting the protein concentration (normalized to 1.0 mg/mL in PBS), 297 μL of the reaction medium was added to a UV–vis microplate, and the absorbance was measured at *λ* = 240 nm for 15 s for 1 min [62].

#### 4.8.5. SOD Activity

The determination of superoxide dismutase (SOD) activity consisted of the quantification of the complex formed between the superoxide anions by the addition of nitroblue tetrazolium (NBT) and hydroxylamine hydrochloride (NH2OH·HCl). The samples were exposed for 2 min to a reaction mixture containing sodium carbonate buffer (50 mM, pH of 10.2), NBT (96 µM), and Triton X-100 (0.6%) with 20 mM NH2OH·HCl. This reaction led to the formation of a yellow coloration and the reduction of NBT, resulting in a blue coloration at *λ* = 560 nm. Measurements were performed every 15 s for 2 min [63].

### 4.9. Gene Expression Analysis

After 48h of transfection, the total RNA was extracted using the PureLink^®^ RNA Mini Kit (Cat. Nº 12183018A; Thermo Scientific, Carlsbad, CA, USA) according to the manufacturer’s instructions. The RNA concentrations were measured with a NanoDrop 2000C (Thermo Fisher Scientific, USA). Subsequently, reverse transcriptase for RT-PCR synthesis was performed using the Superscript III kit (Invitrogen, USA) with the Applied Biosystems™ VeritiPro™ Thermal Cycler, 96-well (Thermo Fisher Scientific, USA). Primers for the target genes were designed based on in silico analyses and the results of functional assays, resulting in the analysis of six target genes (*TP53*, *AIFM1*, *NFE2L2*, *SLC7A11*, *TRFC*, and *ACSL4*) and three non-target genes (*MMP9*, *MMP11*, and *GPX4*) (see Table S3). The qPCR reaction was prepared with a final volume of 11.5 μL, containing 5 pmol of each primer oligonucleotide, 10 ng of cDNA template, and 5 μL of GoTaq qPCR Master Mix, with the remaining volume made up of water. The qPCR was performed using the StepOnePlus™ Real-Time PCR System (4376600, Applied Biosystems, Singapore) under the following cycling conditions: 50 °C for 2 min for UDG incubation and 95 °C for 2 min for enzymatic activation; 40 cycles for 15 min at 90 °C (denaturation), 15 s at 60 °C (annealing/extension) and 1 min at 72 °C (reading); and a melting curve was performed at 95 °C for 15 s, 50 °C for 1 min, and 95 °C for 15 s. The relative expression levels were calculated using the 2^-ΔΔCt^ method proposed by Livak and Schmittgen [64], with *β-actin* as the endogenous control.

### 4.10. Statistical Analysis

Statistical tests were conducted on three biological replicates, with at least three technical replicates for each condition. The Shapiro–Wilk test was used to assess the normality of the data distribution. If the data followed a normal distribution, comparisons were made using an ANOVA followed by Tukey’s post hoc test. Differences in gene expression were analyzed using Student’s t-test to compare the mean relative expression (2^−ΔΔCt^) between the experimental groups and the control. The following significance levels were considered: * *p* < 0.05, ** *p* < 0.01, *** *p* < 0.001, and ** *p* < 0.0001. All the statistical analyses were performed using GraphPad Prism (version 10.0) (GraphPad Software, San Diego, CA, USA).

## 5. Conclusions

Taken together, our findings suggest that miR-25-3p plays a functional role in modulating key features of T24 bladder cancer cells, including cell proliferation, migration, oxidative stress, and viability, by regulating gene expression. The overexpression of miR-25-3p promotes a more aggressive cellular phenotype. An increased migratory capacity was linked to the significant downregulation of *TP53*, *MMP9*, and *MMP11* at the mRNA level, potentially contributing to enhanced metastatic behavior. Additionally, the downregulation of *TFRC*, *ACSL4*, *SLC7A11*, and *SLC3A2*, along with the upregulation of *GPX4*, may contribute to the establishment of a ferroptosis-resistant phenotype. Although our results support a potential oncogenic role for miR-25-3p using an in vitro MIBC model with transfection, the exact mechanisms and direct or indirect gene targets involved are still not fully understood. Nevertheless, these findings underscore the importance of miR-25-3p as a potential therapeutic target in MIBC, highlighting its role in tumor progression and its potential contribution to improved therapeutic outcomes, constituting a starting point for future studies.

## Data Availability

The original contributions presented in this study are included in the article/Supplementary Materials. Further inquiries can be directed to the corresponding author.

## Supplementary Materials

The following supporting information can be downloaded at: https://www.mdpi.com/article/10.3390/ph18091382/s1, Figure S1: Survival analysis applied to miR-25-3p; Figure S2: STR profiles of the T24 cell line; Table S1: Table of miR-25-3p only luciferase validated target genes; Table S2: β coefficients from the adjusted multivariate regression between miR-25-3p and the expression of associated genes in TCGA-BLCA, controlling for CNV and promoter methylation; Table S3: Table of primers used for quantitative PCR.

## Abbreviations

The following abbreviations are used in this manuscript:

2^−ΔΔCt^	2-Delta-delta Ct
μg	Microgram
μL	Microliter
μM	Micromole
ABC	ATP-binding cassette
*ACSL4*	Acyl-CoA synthetase long-chain family member 4
*AIFM1*	Apoptosis-inducing factor mitochondria-associated 1
BCL2L11	BCL2-like 11
BLCA	Bladder cancer
BP	Biological process
CAT	Catalase
CC	Cellular component
cDDP	Cisplatin
CDH1	Cadherin 1
cDNA	Complementary DNA
CDNB	1-Chloro-2,4-dinitrobenzene
DMSO	Dimethylsulfoxide
DTNB	Ellman’s reagent
ECM	Extracellular matrix
EMT	Epithelial–mesenchymal transition
ERBB2	Erb-b2 receptor tyrosine kinase 2
EZH2	Enhancer of zeste 2 polycomb repressive complex 2 subunit
FBS	Fetal bovine serum
FBXW7	F-Box and WD40 domain-containing protein 7 gene
FC	Fold change
FDA	Fluorescein diacetate
FDR	False discovery rate
*FTH1*	Ferritin heavy chain 1
GO	Gene Ontology
*GPX4*	Glutathione peroxidase 4
GR	Glutathione reductase
GSH	Reduced glutathione
GSSG	Oxidized glutathione
GST	Glutathione S-transferase
GT	Glutathione
h	Hour
HO	Hoescht 33342
IN	Inhibitor
KEGG	Kyoto Encyclopedia of Genes and Genomes
KLF4	Kruppel-like factor 4
KM	Kaplan–Meier
*MCM7*	Minichromosome maintenance complex component 7
MDM2	MDM2 proto-oncogene
MF	Molecular function
MI	Mimic
MIBC	Muscle-invasive bladder cancer
min	Minute
miRNA	Micro-RNA
MMP	Matrix metalloproteinase
*MMP9*	Matrix metallopeptidase 9
*MMP11*	Matrix metallopeptidase 11
mRNA	Messenger RNA
MTT	3-(4,5-Dimethylthiazol-2yl)-2,5-di-diphenyltetrazoline bromide
NADP	Nicotinamide-adenine dinucleotide phosphate
NBT	Nitroblue tetrazolium
NC	Negative control
*NFE2L2*	Nuclear factor erythroid 2-related factor 2
NH2OH·HCl	Hydroxylamine hydrochloride
NMIBC	Non-muscle-invasive bladder cancer
PBS	Phosphate-buffered saline
PI	Propidium iodide
pmol	Picomole
PPI	Protein–protein interaction
*PTEN*	Phosphatase and tensin homologue
qPCR	Real-time quantitative PCR
RC	Radical cystectomy
RPM	Revolutions per minute
RPMI 1640	Roswell Park Memorial Institute 1640
RT-PCR	Reverse-transcription polymerase chain reaction
RT-qPCR	Quantitative reverse-transcription PCR
sec	Second
*SLC3A2*	Solute carrier family 3 member 2
*SLC7A10*	Solute carrier family 7 member 10
*SLC7A11*	Solute carrier family 7 member 11
SMAD	Suppressor of mothers against decapentaplegic
SMAD2	Suppressor of mothers against decapentaplegic 2
SMAD3	Suppressor of mothers against decapentaplegic 3
SMAD7	Suppressor of mothers against decapentaplegic 7
SOD	Superoxide dismutase
STR	Short tandem repeat
TBARS	Thiobarbituric acid-reactive substance
TBA	Thiobarbituric acid
TCA	Trichloroacetic acid
TCGA	The Cancer Genome Atlas
TGF-β1	Transforming growth factor β1
*TP53*	Tumor protein P53
*TRFC*	Transferrin receptor
TWIST1	Twist family BHLH transcription factor 1
UBC	Urothelial carcinoma of the bladder
UTR	Untranslated region
