# Supplementary material for: miR-25-3p Modulates Tumor Aggressiveness and Ferroptosis Escape in T24 Bladder Cancer Cells In Vitro
Source: Pharmaceuticals (Basel). 2025 Sep 16;18(9):1382. doi: 10.3390/ph18091382 (PMC12472857; doi:10.3390/ph18091382)
Supplement: Supplementary file 1 [file pharmaceuticals-18-01382-s001.zip › pharmaceuticals-3774402-supplementary.pdf]

pharmaceuticals

MDPI

Article

miR-25-3p Modulates Tumor Aggressiveness and Ferroptosis Escape in T24 Bladder Cancer Cells In Vitro

Andresa Hiromi Sakai [1], Érica Romão Pereira [1], Anna Gabriele Prado dos Santos [1], Débora Hipólito Quadreli [2], Luan Vitor Alves de Lima [1], Diego Luis Ribeiro [3], Samira Rahimirad [4], Carolina Mathias [5], Monyse de Nóbrega [6,7], Mário Sérgio Mantovani [1], Glaura Scantamburlo Alves Fernandes [1,2], Ilce Mara de Syllos Cólus [1] and Juliana Mara Serpeloni [1,*]

1 Post-Graduation Program in Genetics and Molecular Biology, Department of General Biology, State University of Londrina, Londrina 86057-970, Brazil; andresa.hiromi@uel.br (A.H.S.); ericaa.romaopg@uel.br (É.R.P.); annagabriele.prado@uel.br (A.G.P.d.S.); luan.vitorlima@uel.br (L.V.A.d.L.); biomsm@uel.br (M.S.M.); glaura@uel.br (G.S.A.F.); colus@uel.br (I.M.d.S.C.)

2 Post-Graduation Program in Experimental Pathology, Department of Immunology, Parasitology and General Pathology, State University of Londrina, Londrina 86057-970, Brazil; debora.hipolito@uel.br

3 Department of Microbiology, Institute of Biomedical Sciences, University of São Paulo, São Paulo 05508-900, Brazil; diegoluisribeiro@usp.br

4 Urologic Oncology Research Group, Cancer Research Program, Research Institute of the McGill University Health Center (RI-MUHC), Montreal, QC H4A 3J1, Canada; samira.rahimirad@mail.mcgill.ca

5 Post-Graduation Program in Genetics, Department of Genetics, Federal University of Parana, Curitiba 81530-980, Brazil; carolina.mathias@ufpr.br

6 Cancer Research Program, Research Institute of the McGill University Health Centre, Montreal, QC H4A 3J1, Canada; monyse.denobrega@mail.mcgill.ca

7 Department of Pathology, McGill University, Montreal, QC H4A 3J1, Canada

* Correspondence: julianaserpeloni@uel.br; Tel.: +55-43-33715149

Academic Editor: Alfredo Berzal-Herranz

Received: 7 July 2025

Revised: 22 August 2025

Accepted: 12 September 2025

Published: 16 September 2025

Citation: Sakai, A.H.; Pereira, É.R.; Santos, A.G.P.d.; Quadreli, D.H.; Lima, L.V.A.d.; Ribeiro, D.L.; Rahimirad, S.; Mathias, C.; Nóbrega, M.d.; Mantovani, M.S.; et al. miR-25-3p Modulates Tumor Aggressiveness and Ferroptosis Escape in T24 Bladder Cancer Cells In Vitro. *Pharmaceuticals* **2025**, *18*, 1382. https://doi.org/10.3390/ph18091382

Copyright: © 2025 by the authors. Licensee MDPI, Basel, Switzerland. This article is an open access article distributed under the terms and conditions of the Creative Commons Attribution (CC BY) license (https://creativecommons.org/licenses/by/4.0/).

Supplementary Material

Figure S1. Survival analysis applied to miR-25-3p. The high or low miRNA expression is not associated with overall survival in muscle-invasive bladder cancer (MIBC).

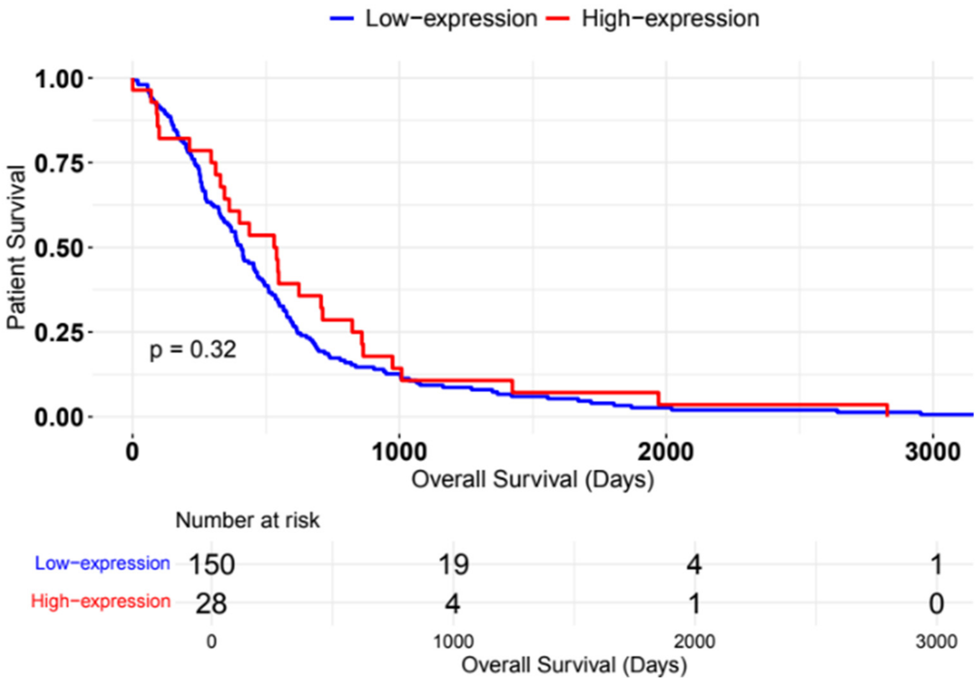

Figure S2: STR profiles of the T24 cell line.

PIPAD
PROGRAMA DE INVESTIGAÇÃO DE PATERNIDADE POR MEIO DE ANÁLISES DE DNA

EXTERNAL STR CELL LINE AUTHENTICATION

SUBMITTER INFORMATION

Name: Juliana M. Serpeloni
Email:
Institution: Universidade Estadual de Londrina
Address: Rod. Celso Garcia Cid, PR445 km 270, Campus Universitário, CCB, LAMON

SAMPLE CHARACTERIZATION

Sample: T24 **Description:** Extracted DNA **Test:** STR-0162024

STR GENOTYPING PROFILE

Locus	Genotype	Locus	Genotype
AMEL	X	D21S11	29
D3S1358	16	D7S820	10, 11
D1S1656	12, 15	D5S818	10, 12
D2S441	11, 15	TPOX	8, 11
D10S1248	14	D8S1179	14
D13S317	12	D12S391	18
Penta E	7, 10	D19S433	13, 14
D16S539	9	SE33	22.2
D18S51	16, 18	D22S1045	16
D2S1338	20, 23	DYS391	-
CSF1PO	10, 12	FGA	22
Penta D	11, 15	DYS576	-
TH01	6	DYS570	-
vWA	17		

TEST DATE

March 30, 2024.

TESTING METHODOLOGY

Testing was performed using the PowerPlex® Fusion 6C System (Promega) on an ABI 3500 Genetic Analyzer (Applied Biosystems). STR profiles were matched using the ATCC Tanabe algorithm (threshold ≥ 56%).

CONCLUSION

95% match with human bladder cancer T-24 (ATCC: HTB-4)

Londrina, April 8, 2025.

Prof. Karen Brajão de Oliveira, PhD.
Coordinator, Paternity Testing Program – DNA Analysis (PIPAD)

Paternity Investigation Program through DNA Analysis
Center for Biological Sciences, State University of Londrina
Rodovia Celso Garcia Cid | PR 445 Km 380 | University Campus | ZIP Code 86057-970 | Londrina - PR, Brazil

Table S1: Table of miR-25-3p only luciferase validated target genes.

Database	mature_mirna_acc	mature_mirna_id	target_symbol	target_entrez	target_ensembl	experiment	support_type
**mirtarbase**	MIMAT0000081	hsa-miR-25-3p	ERBB2	2064	ENSG00000141736	Luciferase reporter assay<br>qRT-PCR<br>Western blot	
**mirtarbase**	MIMAT0000081	hsa-miR-25-3p	ERBB2	2064	ENSG00000141736	Luciferase reporter assay<br>qRT-PCR<br>Western blot	Functional MTI
**mirtarbase**	MIMAT0000081	hsa-miR-25-3p	LATS2	26524	ENSG00000150457	Luciferase reporter assay<br>qRT-PCR<br>Western blot	
**mirtarbase**	MIMAT0000081	hsa-miR-25-3p	LATS2	26524	ENSG00000150457	Luciferase reporter assay<br>qRT-PCR<br>Western blot	Functional MTI
**mirtarbase**	MIMAT0000081	hsa-miR-25-3p	TGFBR2	7048	ENSG00000163513	Luciferase reporter assay<br>qRT-PCR<br>Western blotting	
**mirtarbase**	MIMAT0000081	hsa-miR-25-3p	SMAD5	4090	ENSG00000113658	Immunoprecipitaion (IP)<br>Luciferase reporter assay<br>Western blotting	
**mirtarbase**	MIMAT0000081	hsa-miR-25-3p	DHFR	1719	ENSG00000228716	Luciferase reporter assay<br>qRT-PCR<br>Western blot	Functional MTI
**mirtarbase**	MIMAT0000081	hsa-miR-25-3p	RECK	8434	ENSG00000122707	Luciferase reporter assay<br>qRT-PCR<br>Western blot	
**mirtarbase**	MIMAT0000081	hsa-miR-25-3p	RECK	8434	ENSG00000122707	Luciferase reporter assay<br>qRT-PCR<br>Western blot	Functional MTI

**mirtarbase**	MIMAT0000081	hsa-miR-25-3p	BCL2L11	10018	ENSG00000153094	Luciferase reporter assay<br>Microarray<br>Western blot<br>qRT-PCR<br>Other<br>Flow<br>PAR-CLIP<br>HITS-CLIP
**mirtarbase**	MIMAT0000081	hsa-miR-25-3p	BCL2L11	10018	ENSG00000153094	Luciferase reporter assay<br>Microarray<br>Western blot<br>qRT-PCR<br>Other<br>Flow<br>PAR-CLIP<br>HITS-CLIP
**mirtarbase**	MIMAT0000081	hsa-miR-25-3p	BCL2L11	10018	ENSG00000153094	Luciferase reporter assay<br>Microarray<br>Western blot<br>qRT-PCR<br>Other<br>Flow<br>PAR-CLIP<br>HITS-CLIP
**mirtarbase**	MIMAT0000081	hsa-miR-25-3p	BCL2L11	10018	ENSG00000153094	Luciferase reporter assay<br>Microarray<br>Western blot<br>qRT-PCR<br>Other<br>Flow<br>PAR-CLIPHITS-CLIP

**mirtarbase**	MIMAT0000081	hsa-miR-25-3p	BCL2L11	10018	ENSG00000153094	Luciferase reporter assay<br>Microarray<br>Western blot<br>qRT-PCR<br>Other<br>Flow<br>PAR-CLIP<br>HITS-CLIP
**mirtarbase**	MIMAT0000081	hsa-miR-25-3p	BCL2L11	10018	ENSG00000153094	Luciferase reporter assay<br>Microarray<br>Western blot<br>qRT-PCR<br>Other<br>Flow<br>PAR-CLIP<br>HITS-CLIP
**mirtarbase**	MIMAT0000081	hsa-miR-25-3p	BCL2L11	10018	ENSG00000153094	Luciferase reporter assay<br>Microarray<br>Western blot<br>qRT-PCR<br>Other<br>Flow<br>PAR-CLIP<br>HITS-CLIP
**mirtarbase**	MIMAT0000081	hsa-miR-25-3p	BCL2L11	10018	ENSG00000153094	Luciferase reporter assay<br>Microarray<br>Western blot<br>qRT-PCR<br>Other<br>Flow<br>PAR-CLIP<br>HITS-CLIP
**mirtarbase**	MIMAT0000081	hsa-miR-25-3p	BCL2L11	10018	ENSG00000153094	Luciferase reporter assay

						Microarray<br>Western blot<br>qRT-PCR<br>Other<br>Flow<br>PAR-CLIP<br>HITS-CLIP	
**mirtarbase**	MIMAT0000081	hsa-miR-25-3p	BCL2L11	10018	ENSG00000153094	Luciferase reporter assay<br>Microarray<br>Western blot<br>qRT-PCR<br>Other<br>Flow<br>PAR-CLIP<br>HITS-CLIP	
**mirtarbase**	MIMAT0000081	hsa-miR-25-3p	BCL2L11	10018	ENSG00000153094	Luciferase reporter assay<br>Microarray<br>Western blot<br>qRT-PCR<br>Other<br>Flow<br>PAR-CLIP<br>HITS-CLIP	
**mirtarbase**	MIMAT0000081	hsa-miR-25-3p	BCL2L11	10018	ENSG00000153094	Luciferase reporter assay<br>Microarray<br>Western blot	Functional MTI
**mirtarbase**	MIMAT0000081	hsa-miR-25-3p	BCL2L11	10018	ENSG00000153094	Luciferase reporter assay<br>Microarray<br>qRT-PCR<br>Western blot	Functional MTI
**mirtarbase**	MIMAT0000081	hsa-miR-25-3p	BCL2L11	10018	ENSG00000153094	Luciferase reporter assay<br>qRT-PCR<br>Western blot	Functional MTI

**mirtarbase**	MIMAT0000081	hsa-miR-25-3p	BCL2L11	10018	ENSG00000153094	Flow<br>Luciferase reporter assay<br>qRT-PCR<br>Western blot	Functional MTI
**mirtarbase**	MIMAT0000081	hsa-miR-25-3p	EZH2	2146	ENSG00000106462	Luciferase reporter assay<br>qRT-PCR<br>Western blot	
**mirtarbase**	MIMAT0000081	hsa-miR-25-3p	EZH2	2146	ENSG00000106462	Luciferase reporter assay<br>qRT-PCR<br>Western blot	Functional MTI
**mirtarbase**	MIMAT0000081	hsa-miR-25-3p	TP53	7157	ENSG00000141510	Luciferase reporter assay<br>qRT-PCR<br>Western blot<br>Other	
**mirtarbase**	MIMAT0000081	hsa-miR-25-3p	TP53	7157	ENSG00000141510	Luciferase reporter assay<br>qRT-PCR<br>Western blot	Functional MTI
**mirtarbase**	MIMAT0000081	hsa-miR-25-3p	CDH1	999	ENSG00000039068	Luciferase reporter assay<br>qRT-PCR<br>Western blot	
**mirtarbase**	MIMAT0000081	hsa-miR-25-3p	CDH1	999	ENSG00000039068	Luciferase reporter assay<br>qRT-PCR<br>Western blot	Functional MTI
**mirtarbase**	MIMAT0000081	hsa-miR-25-3p	KLF4	9314	ENSG00000136826	Western blot<br>Other<br>Luciferase reporter assay	
**mirtarbase**	MIMAT0000081	hsa-miR-25-3p	KLF4	9314	ENSG00000136826	Western blot<br>Other<br>Luciferase reporter assay	
**mirtarbase**	MIMAT0000081	hsa-miR-25-3p	KLF4	9314	ENSG00000136826	Luciferase reporter assay	Functional MTI
**mirtarbase**	MIMAT0000081	hsa-miR-25-3p	FBXW7	55294	ENSG00000109670	Luciferase reporter assay	

						qRT-PCR<br>Western blot<br>Immunofluorescence<br>Immunohistochemistry	
**mirtarbase**	MIMAT0000081	hsa-miR-25-3p	FBXW7	55294	ENSG00000109670	Luciferase reporter assay<br>qRT-PCR<br>Western blot<br>Immunofluorescence<br>Immunohistochemistry	
**mirtarbase**	MIMAT0000081	hsa-miR-25-3p	FBXW7	55294	ENSG00000109670	Luciferase reporter assay<br>qRT-PCR<br>Western blot<br>Immunofluorescence<br>Immunohistochemistry	
**mirtarbase**	MIMAT0000081	hsa-miR-25-3p	FBXW7	55294	ENSG00000109670	Luciferase reporter assay<br>qRT-PCR<br>Western blot<br>Immunofluorescence<br>Immunohistochemistry	
**mirtarbase**	MIMAT0000081	hsa-miR-25-3p	FBXW7	55294	ENSG00000109670	Luciferase reporter assay<br>qRT-PCR<br>Western blot<br>Immunofluorescence<br>Immunohistochemistry	
**mirtarbase**	MIMAT0000081	hsa-miR-25-3p	FBXW7	55294	ENSG00000109670	Luciferase reporter assay<br>qRT-PCR<br>Western blot	Functional MTI
**mirtarbase**	MIMAT0000081	hsa-miR-25-3p	FBXW7	55294	ENSG00000109670	Luciferase reporter assay<br>Western blot	Functional MTI
**mirtarbase**	MIMAT0000081	hsa-miR-25-3p	ATP2A2	488	ENSG00000174437	In situ hybridization<br>Luciferase reporter assay<br>qRT-PCR	

						Western blot	
**mirtarbase**	MIMAT0000081	hsa-miR-25-3p	ATP2A2	488	ENSG00000174437	In situ hybridization<br>Luciferase reporter assay<br>qRT-PCR<br>Western blot	Functional MTI
**mirtarbase**	MIMAT0000081	hsa-miR-25-3p	DSC2	1824	ENSG00000134755	Luciferase reporter assay<br>Western blot	
**mirtarbase**	MIMAT0000081	hsa-miR-25-3p	DSC2	1824	ENSG00000134755	Luciferase reporter assay<br>Western blot	Functional MTI
**mirtarbase**	MIMAT0000081	hsa-miR-25-3p	SMAD7	4092	ENSG00000101665	Luciferase reporter assay<br>qRT-PCR<br>Western blot	
**mirtarbase**	MIMAT0000081	hsa-miR-25-3p	SMAD7	4092	ENSG00000101665	Luciferase reporter assay<br>qRT-PCR<br>Western blot	
**mirtarbase**	MIMAT0000081	hsa-miR-25-3p	SMAD7	4092	ENSG00000101665	Luciferase reporter assay	Functional MTI
**mirtarbase**	MIMAT0000081	hsa-miR-25-3p	SMAD7	4092	ENSG00000101665	Luciferase reporter assay<br>qRT-PCR<br>Western blot	Functional MTI
**mirtarbase**	MIMAT0000081	hsa-miR-25-3p	MDM2	4193	ENSG00000135679	Luciferase reporter assay<br>qRT-PCR<br>Western blot<br>PAR-CLIP	
**mirtarbase**	MIMAT0000081	hsa-miR-25-3p	MDM2	4193	ENSG00000135679	Luciferase reporter assay<br>qRT-PCR<br>Western blot<br>PAR-CLIP	
**mirtarbase**	MIMAT0000081	hsa-miR-25-3p	MDM2	4193	ENSG00000135679	Luciferase reporter assay<br>qRT-PCR<br>Western blot	Functional MTI
**mirtarbase**	MIMAT0000081	hsa-miR-25-3p	MTF1	4520	ENSG00000188786	Luciferase reporter assay	

						qRT-PCR<br>Western blot<br>Immunocytochemistry (ICC)	
**mirtarbase**	MIMAT0000081	hsa-miR-25-3p	REV3L	5980	ENSG00000009413	Luciferase reporter assay<br>PAR-CLIP	
**mirtarbase**	MIMAT0000081	hsa-miR-25-3p	REV3L	5980	ENSG00000009413	Luciferase reporter assay<br>PAR-CLIP	
**mirtarbase**	MIMAT0000081	hsa-miR-25-3p	REV3L	5980	ENSG00000009413	Luciferase reporter assay	Functional MTI
**mirtarbase**	MIMAT0000081	hsa-miR-25-3p	RGS3	5998	ENSG00000138835	Luciferase reporter assay<br>qRT-PCR<br>Western blot	Functional MTI
**mirtarbase**	MIMAT0000081	hsa-miR-25-3p	KAT2B	8850	ENSG00000114166	Western blot<br>Luciferase reporter assay<br>Other	
**mirtarbase**	MIMAT0000081	hsa-miR-25-3p	KAT2B	8850	ENSG00000114166	Western blot<br>Luciferase reporter assay	Functional MTI
**mirtarbase**	MIMAT0000081	hsa-miR-25-3p	TCEAL1	9338	ENSG00000172465	Flow<br>Luciferase reporter assay<br>qRT-PCR<br>Western blot	
**mirtarbase**	MIMAT0000081	hsa-miR-25-3p	TCEAL1	9338	ENSG00000172465	Flow<br>Luciferase reporter assay<br>qRT-PCR<br>Western blot	Functional MTI
**mirtarbase**	MIMAT0000081	hsa-miR-25-3p	HAND2	9464	ENSG00000164107	Luciferase reporter assay	
**mirtarbase**	MIMAT0000081	hsa-miR-25-3p	HAND2	9464	ENSG00000164107	Luciferase reporter assay	Functional MTI
**mirtarbase**	MIMAT0000081	hsa-miR-25-3p	CCL26	10344	ENSG00000006606	Luciferase reporter assay<br>qRT-PCR<br>Western blot	

**mirtarbase**	MIMAT0000081	hsa-miR-25-3p	CCL26	10344	ENSG00000006606	Luciferase reporter assay<br>qRT-PCR<br>Western blot	Functional MTI
**mirtarbase**	MIMAT0000081	hsa-miR-25-3p	WDR4	10785	ENSG00000160193	Luciferase reporter assay<br>qRT-PCR<br>Western blot	
**mirtarbase**	MIMAT0000081	hsa-miR-25-3p	WDR4	10785	ENSG00000160193	Luciferase reporter assay<br>qRT-PCR<br>Western blot	Functional MTI
**mirtarbase**	MIMAT0000081	hsa-miR-25-3p	SEMA4C	54910	ENSG00000168758	Luciferase reporter assay<br>qRT-PCR<br>Western blot	Functional MTI
**mirtarbase**	MIMAT0000081	hsa-miR-25-3p	TWIST1	7291	ENSG00000122691	Luciferase reporter assay<br>qRT-PCR<br>Western blot	
**mirtarbase**	MIMAT0000081	hsa-miR-25-3p	CYP2B6	1555	ENSG00000197408	EMSA<br>Luciferase reporter assay<br>qRT-PCR<br>Western blot	Functional MTI
**mirtarbase**	MIMAT0000081	hsa-miR-25-3p	CDKN1C	1028	ENSG00000273707	Luciferase reporter assay<br>qRT-PCR<br>Western blot<br>Other	
**mirtarbase**	MIMAT0000081	hsa-miR-25-3p	CDKN1C	1028	ENSG00000273707	Luciferase reporter assay<br>qRT-PCR<br>Western blot	Functional MTI
**mirtarbase**	MIMAT0000081	hsa-miR-25-3p	CPEB1	64506	ENSG00000277445	Luciferase reporter assay	
**mirtarbase**	MIMAT0000081	hsa-miR-25-3p	CPEB1	64506	ENSG00000277445	Luciferase reporter assay	Functional MTI
**mirtarbase**	MIMAT0000081	hsa-miR-25-3p	PTEN	5728	ENSG00000284792	Luciferase reporter assay<br>qRT-PCR<br>Western blot	

						Microarray	
**mirtarbase**	MIMAT0000081	hsa-miR-25-3p	PTEN	5728	ENSG00000284792	Luciferase reporter assay<br>qRT-PCR<br>Western blot<br>Microarray	
**mirtarbase**	MIMAT0000081	hsa-miR-25-3p	PTEN	5728	ENSG00000284792	Luciferase reporter assay	Functional MTI
**mirtarbase**	MIMAT0000081	hsa-miR-25-3p	CBR3-AS1	100506428	ENSG00000236830	Immunohistochemistry (IHC)<br>Luciferase reporter assay<br>qRT-PCR<br>Western blot	
**tarbase**	MIMAT0000081	hsa-miR-25-3p	CDKN1C	1028	ENSG00000129757	HITS-CLIP<br>Luciferase Reporter Assay	negative
**tarbase**	MIMAT0000081	hsa-miR-25-3p	BCL2L11	10018	ENSG00000153094	PAR-CLIP<br>HITS-CLIP<br>Luciferase Reporter Assay<br>Chimeric fragments	negative
**tarbase**	MIMAT0000081	hsa-miR-25-3p	FBXW7	55294	ENSG00000109670	PAR-CLIP<br>HITS-CLIP<br>Luciferase Reporter Assay	negative
**tarbase**	MIMAT0000081	hsa-miR-25-3p	MDM2	4193	ENSG00000135679	PAR-CLIP<br>HITS-CLIP<br>qCLASH<br>Luciferase Reporter Assay<br>Chimeric fragments	negative
**tarbase**	MIMAT0000081	hsa-miR-25-3p	TSC1	7248	ENSG00000165699	HITS-CLIP<br>Luciferase Reporter Assay	negative
**tarbase**	MIMAT0000081	hsa-miR-25-3p	KAT2B	8850	ENSG00000114166	HITS-CLIP<br>Luciferase Reporter Assay	negative

Table S2: β coefficients from the adjusted multivariate regression between miR-25-3p and the expression of associated genes in TCGA-BLCA, controlling for CNV and promoter methylation. Genes with an adjusted p-value < 0.05 (Benjamini-Hochberg FDR) were considered significant. The direction of association indicates whether mRNA expression increases (↑) or decreases (↓) with increasing miRNA expression.

**Gene**	**β coefficients (miR-25-3p)**	**p-value**	**Adjusted p-value**	**Direction of association**
SLC3A2	-0,185	$4{,}99 \times 10^{-5}$	$4{,}59 \times 10^{-4}$	Negative (↓)
SLC7A10	0,588	$2{,}80 \times 10^{-6}$	$6{,}45 \times 10^{-5}$	Positive (↑)

Table S3: Table of primers used for quantitative PCR. The name, primer's sequences, amplicon size, percentage of GC, and annealing temperature are reported for each gene.

Gene	Sequence 5′-3′	Amplicon size	%GC	TºC
β-actin	F: CCCTCCATCGTCCACCGC	200	72.22	62.53
	R: CTGCTGTCACCTTCACCGT		57.89	59.93
Acyl-CoA Synthetase Long Chain Family Member 4 (*ACSL4*)	F: ATAAAGCAGAGTACCCTGAAG	80	42.86	54.73
	R: CAAGTTTTCTGGGTTAGATCC		42.86	54.18
Apoptosis-Inducing Factor Mitochondria-Associated 1 (*AIFM1*)	F: CGATATAAAGTTGGGAAGGAG	123	42.86	53.52
	R: CTCCAGAACATTGACTGATG		45.00	53.72
Glutathione Peroxidase 4 (*GPX4*)	F: GAAGTAAACTACACTCAGCTC	126	42.86	53.74
	R: CTCTTTGATCTCTTCGTTACTC		40.91	54.12
Matrix Metalloproteinase 9 (*MMP9*)	F: CACACGCCTTTCCTCCTG	88	61.11	58.03
	R: ACCACGACGCCCTTGCC		70.59	62.80
Matrix Metalloproteinase 11 (*MMP11*)	F: GATAGACACCAATGAGATTGC	109	42.9	54.4
	R: TTTGAAGAAAAAGAGCTCGC		40.0	54.9
Nuclear Factor Erythroid 2-Related Factor 2 (*NFE2L2*)	F: CGTTTGTAGATGACAATGAGG	122	42.86	54.71
	R: AGAAGTTTCAGGTGACTGAG		45.00	54.64
Solute Carrier Family 3 Member 2 (*SLC3A2*)	F: CCAGAAGGATGATGTCGCTCAG	128	54.55	60.80
	R: GAGTAAGGTCCAGAATGACACGG		52.17	60.68
Solute Carrier Family 7 Member 11 (*SLC7A11*)	F: TTTGCACCCTTTGACAATGA	184	40.00	56.05
	R: GGAAAACAAAGCTGGGATGA		45.00	56.21
Transferrin Receptor (*TRFC*)	F: AAGATTCAGGTCAAAGACAG	108	40.00	52.82
	R: CTTACTATACGCCACATAACC		42.86	53.66
Tumor Protein P53 (*TP53*)	F: CCATCCACTACAACTACAT	136	42.11	51.22
	R: GCACAAACACGCACCTC		58.82	56.89
